# Supplementary material for: Surfactin and Capric Acid Affect the Posaconazole Susceptibility of Candida albicans Strains with Altered Sterols and Sphingolipids Biosynthesis
Source: Int J Mol Sci. 2023 Dec 15;24(24):17499. doi: 10.3390/ijms242417499 (PMC10743603; doi:10.3390/ijms242417499)
Supplement: Supplementary file 1 [file ijms-24-17499-s001.zip › ijms-2727071-supplementary.pdf]

Supplementary Material

The Table S1 represent the MIC50 values of PSZ determined for *C. albicans* strains which were used in this study.

Table S1. MIC50 values of PSZ determined for *C. albicans* strains with altered ergosterol (SC5314 (WT), KS058, 10C1B1I1, 27A5A33A and 9B4B34A; figure 7A) or sphinoglipids biosynthesis SN95 (WT), *fen1Δ/Δ*, *fen12Δ/Δ* and *fen1Δ/Δ*; *fen12Δ/Δ*) in presence of SU (4 - 32 µg/mL) or CA (11.35 – 90.60 µg/mL) or for PSZ alone. The experiment was performer according to method described in section 4.3. and the MIC50 values was determined using data presented in figures 3, 4, 5 and 6.

***C. albicans* strain**	**PSZ alone [µg/mL]**	**+ SU [4 µg/mL]**	**+ SU [8 µg/mL]**	**+ SU [16 µg/mL]**	**+ SU [32 µg/mL]**	**+ CA [11.35 µg/mL]**	**+ CA [22.70 µg/mL]**	**+ CA [45.30 µg/mL]**	**+ CA [90.60 µg/mL]**
SC5314	0.0156	0.0313	0.0156	0.0156	0.0156	0.0156	0.0078	0.0078	0.0000
KS058	ND	ND	ND	ND	0.0000	0.0000	0.0000	0.0000	0.0000
10C1B1I1	0.0156	0.0313	0.0156	0.0156	0.0156	0.0078	0.0078	0.0078	0.0078
27A5A33A	ND	0.0313	0.0313	0.0313	0.0313	0.0078	0.0078	0.0156	0.0156
9B4B34A	0.0156	0.0313	0.0078	0.0156	0.0156	0.0078	0.0078	0.0078	0.0078
SN95	0.0156	0.0078	0.0078	0.0078	0.0078	0.0078	0.0078	0.0078	0.0156
fen1Δ/Δ	0.0156	0.0156	0.0156	0.0156	0.0156	0.0156	0.0078	0.0078	0.0078
fen12Δ/Δ	0.0156	0.0156	0.0156	0.0156	0.0156	0.0156	0.0078	0.0078	0.0000
fen1Δ/Δ; *fen12Δ/Δ*	0.0078	0.0078	0.0078	0.0078	0.0039	0.0000	0.0000	0.0000	0.0000
